# Age-dependent health risks of smoking: greater harm associated with initiation before alveolar maturation (age 24)

**DOI:** 10.7189/jogh.16.04215

**Published:** 2026-08-28

**Authors:** Ziyi Liu, Zhiqi Zhao, Yutong Xiao, Ruoyan Xiong, Huiru Zheng, Yifan Liu, Qixuan Huang, Rui Zhao, Junxia Yan, Yan Chen, Huihui Zeng

**Affiliations:** 1 Department of Pulmonary and Critical Care Medicine, The Second Xiangya Hospital, Central South University, Changsha, Hunan, China; 2 Research Unit of Respiratory Disease, Central South University, Changsha, Hunan, China; 3 Clinical Medical Research Center for Pulmonary and Critical Care Medicine in Hunan Province, Changsha, Hunan, China; 4 Diagnosis and Treatment Center of Respiratory Disease in Hunan Province, Changsha, Hunan, China; 5 Tianjin Medical University, Tianjin, China; 6 Department of Epidemiology and Health Statistics, XiangYa School of Public Health, Central South University, Changsha, Hunan, China

**Keywords:** UK Biobank, NHANES, alveolar development stage, COPD

## Abstract

**Background:**

Smoking during the alveolar developmental stage (ADS) (≤24 years) may increase cardiopulmonary disease risk beyond the effects of cumulative exposure, but these interactions remain unclear. We aimed to determine whether smoking initiation during ADS modifies the associations between cumulative smoking exposure and cardiopulmonary disease, multimorbidity, and all-cause mortality.

**Methods:**

We analysed 15,077 participants from the US National Health and Nutrition Examination Survey and two UK Biobank cohorts: 121,852 individuals in the disease cohort and 136,528 in the mortality cohort. Multivariable logistic regression and Cox proportional hazards models examined associations of smoking initiation timing (≤24 *vs. *>24 years), cumulative exposure (>20 *vs.* ≤20 pack-years), and their interaction with chronic obstructive pulmonary disease (COPD), cardiovascular disease, all-cause mortality, and multimorbidity. We assessed both multiplicative and additive interactions. We also evaluated an ordinal exposure score combining initiation timing and pack-years. Zero-inflated negative binomial regression quantified comorbidity burden, and stratified analyses assessed the effects of smoking cessation duration.

**Results:**

Smoking initiation in the ADS amplified smoking-related disease risk. For COPD, ADS initiators with high cumulative exposure had a greater risk (hazard ratio (HR) = 3.45; 95% confidence interval (CI) = 3.29, 3.62) than post-ADS initiators with high exposure (HR = 2.33; 95% CI = 1.91, 2.83; *P* for multiplicative interaction <0.001). Additive interaction was also observed, with a relative excess risk due to interaction of 0.43 (95% CI = 0.08, 0.79) and an attributable proportion of 0.16 (95% CI = 0.02, 0.29). Each one-point increase in the exposure score was associated with higher COPD risk (HR = 2.29; 95% CI = 2.20, 2.38) and mortality (HR = 1.39; 95% CI = 1.36, 1.43), with 82.1% of COPD burden attributable to score ≥1 exposure. High exposure was associated with a larger reduction in comorbidity-free probability among ADS initiators (OR = 0.55) than post-ADS initiators (OR = 0.65) (*P* for interaction <0.001). Smoking cessation reduced COPD and mortality risks in both groups, with greater relative reductions among ADS initiators.

**Conclusions:**

Smoking initiation during ADS, combined with greater cumulative exposure, is associated with increased risks of COPD, cardiovascular morbidity, multimorbidity, and all-cause mortality. Incorporating developmental timing may improve smoking-related risk stratification.

Cigarette smoking persists as a leading contributor to the global burden of disease, driving substantial mortality, disability, and economic costs. According to the Global Burden of Disease Study, smoking was directly responsible for 7.69 million deaths and 200 million disability-adjusted life years lost, representing the leading cause of death among men and accounting for 20.2% of male mortality [1]. Cumulative smoking exposure, quantified as pack-years, is a well-established core risk factor for chronic obstructive pulmonary disease (COPD), pulmonary fibrosis, cardiovascular disease (CVD), and various cancer types [2–8].

Emerging evidence suggests that the age of smoking initiation itself may constitute an independent risk factor. Individuals initiating smoking before age 15 exhibit a 41% higher risk of developing COPD compared to those starting after age 15 – a risk not fully explained by cumulative lifetime exposure – implying heightened vulnerability during critical periods of lung development [9]. Early initiation is further associated with increased risks of overall CVD, coronary artery disease, stroke, and interacts with genetic susceptibility to amplify respiratory cancer incidence [10,11]. Human lung development begins *in utero* and continues through childhood, adolescence, and early adulthood. This developmental continuum includes ongoing alveolar formation and maturation, while lung function follows trajectories that generally reach their peak in early adulthood rather than at a single universally defined age [12–15]. Building on our prior work, we use the term alveolar developmental stage (ADS) to describe this vulnerable developmental window and, for analytic purposes, define it as spanning birth to approximately 24 years of age. We acknowledge that this age boundary is a literature-informed operational cutoff rather than a precise biological threshold. Tobacco exposure during the ADS may therefore have disproportionate long-term consequences. In our previous study, COPD patients who smoked during this period experienced significantly worse clinical outcomes [16]. Concurrently, individuals who smoked during the ADS generally exhibit a greater comorbidity burden and poorer overall health status [17].

Crucially, no studies have systematically evaluated the joint effects of smoking initiation timing and cumulative pack-year exposure on later health outcomes. To address this gap, we leveraged two complementary population-based resources with distinct analytic purposes. We used the US National Health and Nutrition Examination Survey (NHANES) to assess cross-sectional associations of smoking initiation during the ADS (≤24 years) and cumulative smoking exposure with prevalent disease and comorbidity burden, whereas the UK Biobank (UKB) was used to evaluate prospective associations with incident cardiopulmonary outcomes and all-cause mortality. We also developed a novel smoking exposure score to quantify the combined influence of smoking initiation timing and cumulative exposure. In addition, we conducted stratified analyses within the UKB to examine whether the association between smoking cessation duration and the risks of COPD and all-cause mortality differed according to smoking initiation during *vs.* after the ADS.

## METHODS

We conducted secondary analyses of two large, publicly available datasets: NHANES and the UKB. We confirm that our reporting adheres to the Journal of Global Health’s GRABDROP guidelines (Table S1 in the **Online Supplementary Document**) [18].

### Study population

For the NHANES cohort, we used data from the 2007–2012 cycles, which employed a multistage, stratified probability sampling design [19]. We restricted our analysis to adult participants aged ≥20 years. We excluded participants with missing data for key variables, including COPD, chronic kidney disease (CKD), hypertension, diabetes, coronary heart disease (CHD), myocardial infarction, stroke, angina, fibrosis-4 index, and smoking metrics, resulting in a final analytical sample of 15,077 participants (Figure S1 in the **Online Supplementary Document**).

The UKB cohort is a large-scale prospective resource that recruited over 500,000 individuals aged 40–69 years across England, Scotland, and Wales, collecting extensive baseline data through touchscreen questionnaires, physical measurements, and biological samples [20]. We excluded participants with baseline diagnoses of stroke, myocardial infarction, myocardial ischaemia, or COPD. We also excluded individuals with missing smoking initiation age, never-smokers, lost-to-follow-up participants, and those lacking data on alcohol consumption, Townsend Deprivation Index, ambient air pollution exposure, body mass index (BMI), or smoking pack-years. After applying these stringent exclusion criteria, 121,852 participants were included in the final disease cohort analysis. Correspondingly, 136,528 individuals were enrolled in the final mortality cohort analysis (Figures S2 and S3 in the **Online Supplementary Document**).

### Study definitions and outcome ascertainment

#### NHANES disease definitions

The definition of COPD was based on post-bronchodilator forced expiratory volume in one second/forced vital capacity of <0.7, physician-diagnosed emphysema self-report, or age >40 years with chronic bronchitis self-report/smoking history concurrent with COPD medication [21]. Hypertension was defined by average systolic blood pressure ≥130 mm Hg, average diastolic blood pressure ≥80 mm Hg, self-reported history, or current antihypertensive medication. Diabetes mellitus was defined by fasting plasma glucose >7 mmol/L, random plasma glucose >11 mmol/L, OGTT 2-hour plasma glucose >11.1 mmol/L, HbA1c >6.5%, self-reported diagnosis, or hypoglycaemic medication use. Additionally, CKD was defined as estimated glomerular filtration rate <60 mL/min/1.73m^2^ or urinary albumin-to-creatinine ratio >30 mg/g. Lastly, CVDs (angina, CHD, myocardial infarction, stroke) were defined by self-reported physician diagnosis, and liver cirrhosis by fibrosis-4 index >1.45.

#### UKB outcome ascertainment

We used the UKB algorithmic approach to ascertain study endpoints, including COPD, myocardial infarction, stroke, chronic ischaemic heart disease, and the secondary outcome of all-cause mortality. Myocardial infarction, COPD, and stroke cases were identified through the earliest recorded instance in hospital inpatient data (ICD-9/ICD-10 codes), death registry records, or baseline/follow-up self-reported diagnoses. Chronic ischaemic heart disease was defined using the earliest occurrence in primary care records, hospital admissions, death registries, or self-reported data with corresponding ICD-10 codes. All-cause mortality data were obtained from the National Health Service registries (England/Wales) and the National Health Service Central Register (Scotland). Participant follow-up extended from enrolment until the first occurrence of the target disease, death, or study end – whichever occurred first [22]. The Charlson Comorbidity Index (CCI) was calculated based on the ICD-10 codes for constituent conditions, with each comorbidity assigned established weights (scores of 1–6); the composite score represented the sum of these weighted components [23].

#### Independent variable

The primary independent variable in this study was age at smoking initiation, categorised into two groups defined by ADS: individuals initiating regular smoking at ≤24 years of age (ADS) and those initiating >24 years of age (post-ADS). This standardised age-based classification was uniformly implemented across both the UKB and NHANES.

#### Covariates

The NHANES covariates included age, sex, race/ethnicity, education, marital status, poverty-to-income ratio (low: ≤1, middle: 1–3, high: >3), BMI (kg/m^2^), alcohol consumption, smoking index (cigarettes/d × smoking years), and disease status (COPD, hypertension, diabetes, CKD, CVD, liver cirrhosis), with relevant disease exclusions during model fitting. The UKB covariates comprised sex, age, smoking pack-years (0–20, >20), educational attainment, alcohol consumption status, BMI, ambient pollutants (nitrogen dioxide and particulate matter levels in 2010), hypertension, diabetes, CKD, and the Townsend Deprivation Index.

#### Statistical analysis

We employed a multi-database analytical framework combined with specific research methodologies. We conducted comparisons between participants who started smoking during the ADS and those who started post-ADS using the Wilcoxon rank-sum test for continuous variables and the χ^2^ test for categorical variables.

Leveraging NHANES cross-sectional data, we constructed multivariable logistic regression models with never-smokers as the reference group. Odds ratios (OR) and 95% confidence intervals (CIs) were estimated for the association between ADS smoking initiation *vs.* post-ADS initiation and disease risk. We adjusted the models for age, sex, race/ethnicity, education, marital status, poverty-to-income ratio, BMI, alcohol consumption, and the smoking index (cigarettes/d × smoking years). In the NHANES analysis, specific survey weights were applied to account for the complex sampling design.

We pre-specified the 24-year cutoff distinguishing smoking initiation during the alveolar developmental period based on biological considerations (completion of active alveolarization) and our prior research framework. Candidate thresholds (15, 19, and 24 years) were compared based on risk differences and interaction significance to identify an operationally useful cutoff. To further address potential concerns about threshold selection, we additionally modelled smoking initiation age as a continuous variable in sensitivity analyses using multivariable Cox proportional hazards regression. Subsequently, we employed Cox proportional hazards models incorporating multiplicative interaction terms to assess the interaction effect between smoking initiation during ADS and cumulative pack-years (>20 *vs.* ≤20 pack-years) on the risks of COPD, cardiovascular outcomes, and all-cause mortality. All interaction analyses between initiation age and cumulative exposure – including the >20 *vs.* ≤20 pack-years comparison – were conducted exclusively in the UKB cohort. To comprehensively evaluate the interaction effect on both multiplicative and additive scales, we assessed multiplicative interaction *via* the cross-product term in Cox models and additive interaction using the relative excess risk due to interaction (RERI) and attributable proportion (AP), with 95% CIs estimated *via* the delta method. We present fully adjusted models, including baseline comorbidities, as our primary analysis, while minimally adjusted models excluding baseline comorbidities are reported as sensitivity analyses to assess the potential impact of overadjustment for possible mediators.

We quantified the impact of pack-years on the CCI severity (expressed as incidence rate ratios) and the probability of being comorbidity-free (expressed as ORs) using zero-inflated negative binomial regression models, stratified by smoking initiation age. We used the zero-inflated negative binomial model solely to handle the excess zero counts in the CCI distribution; the ‘structural zero’ component is a statistical construct and does not represent a clinically defined latent class. We constructed a novel ordinal smoking exposure score (range 0–3) integrating smoking initiation age (≤24 years/>24 years) and pack-years (≤20/>20). The smoking exposure score was developed based on our prior research, which identified significant interactions between the age of smoking initiation and cumulative pack-years, particularly during the alveolar development stage (≤24 years). These findings indicated that early smoking initiation, even at lower cumulative exposure levels, results in substantially higher long-term risks of respiratory diseases. The score categories were thus designed to reflect these compounded risks, with higher scores assigned to individuals who began smoking during this critical developmental window and accumulated greater exposure. We then used the Cox proportional hazards models to estimate the hazard ratio (HR) per unit increase in this score and the risk differences between score categories. We calculated the population attributable fraction to evaluate the disease burden contribution of different score levels to COPD and mortality.

Stratified Cox proportional hazards models assessed the gradient effect of smoking cessation duration (≤5, 6–10, >10 years) on COPD incidence risk and mortality, while also testing the interaction with smoking initiation during the alveolar developmental period. We evaluated model performance using time-dependent receiver operating characteristic curves, decision curve analysis, and the construction of risk prediction nomograms.

We used *R*, version 4.2.1 (R Core Team, Vienna, Austria) for all analyses.

## RESULTS

### Participant characteristics and threshold validation

The 24-year threshold defining ADS smoking was biologically grounded in peak lung function attainment (25 years) and statistically optimised; among candidate cutoffs (15, 19, 24 years), 24 years maximised COPD risk differences (ΔHR = 1.12) with strongest interaction significance (*P* for interaction <0.001) (Table S2 in the **Online Supplementary Document**). In sensitivity analyses treating smoking initiation age as a continuous variable, each one-year increase in initiation age was associated with a reduced COPD risk in both the basic model (HR = 0.990; 95% CI = 0.985, 0.995) and the fully adjusted model (HR = 0.990; 95% CI = 0.984, 0.995) (Table S3 in the **Online Supplementary Document**). Analysis of NHANES participants revealed that individuals initiating smoking during alveolar development exhibited higher cumulative smoking exposure (mean smoking index: 394.3 *vs.* 209.4; *P* < 0.001), lower socioeconomic status (*e.g.* above high school education: 50.9% *vs.* 54.2%), and distinct male predominance (56.7% *vs.* 45.3%) than those initiating smoking post-ADS (Table S4 in the **Online Supplementary Document**). In the UKB cohort, ADS initiators similarly demonstrated elevated smoking burden (>20 pack-years: >45% *vs.* <23%; *P* < 0.001) and greater socioeconomic deprivation (Townsend index: −1.8 *vs.* −0.9; *P* < 0.001) than post-ADS initiators (Tables S5–6 in the **Online Supplementary Document**). Additionally, there was a strikingly divergent sex distribution, with more male ADS initiators (51.1–53.1%) and more female post-ADS initiators (59.8–61.2%) (*P* < 0.001). These robust, cohort-consistent patterns associate early smoking initiation with heavier lifelong exposure, socioeconomic disadvantage, and sex-specific susceptibility.

### Disease risks by smoking initiation age

The NHANES database analysis showed that individuals who initiated smoking during the ADS had a higher risk of COPD (OR = 3.48; 95% CI = 2.72, 4.44; *P* < 0.001), followed by myocardial infarction (OR = 1.48; 95% CI = 1.05, 2.08; *P* = 0.026) and stroke (OR = 1.41; 95% CI = 1.06, 1.86; *P* = 0.018) compared to never-smokers (Table S7 in the **Online Supplementary Document**). Notably, among those who initiated smoking post-ADS, only CHD risk was significantly elevated compared with never-smokers (OR = 1.91; 95% CI = 1.01, 3.62; *P* = 0.047). No statistically significant differences in the risks of CKD, diabetes, hypertension, or liver cirrhosis were observed across the studied groups. Given the cross-sectional nature of the NHANES data, these findings prompted a prospective examination of the associations in the UKB cohort.

### Synergistic amplification of disease and mortality risks

We examined multiplicative and additive interactions between ADS initiation (≤24 years) and high cumulative exposure (>20 pack-years) for each outcome (**Table 1**). The strongest evidence of interaction was observed for COPD. On the multiplicative scale, the interaction term was highly significant (*P* < 0.001), with the ADS/high-exposure group having a higher risk (HR = 3.45; 95% CI = 3.29, 3.62) compared to the post-ADS/high-exposure group (HR = 2.33; 95% CI = 1.91, 2.83). On the additive scale, the RERI was 0.43 (95% CI = 0.08, 0.79), and the AP was 0.16 (95% CI = 0.02, 0.29), indicating that the combined effect exceeded the sum of individual effects. For chronic myocardial ischaemia, multiplicative interaction was significant (*P* = 0.010), and additive measures also suggested interaction, with an RERI of 0.19 (95% CI = 0.00–0.38) and an AP of 0.17 (95% CI = 0.00, 0.34), though the magnitude was smaller than for COPD. The multiplicative interaction was also significant for all-cause mortality (*P* < 0.001), but the additive measures did not reach statistical significance – RERI was 0.15 (95% CI = –0.05, 0.34) and AP was 0.09 (95% CI = –0.03, 0.22). No significant multiplicative or additive interactions were observed for stroke or acute myocardial infarction. Minimally adjusted models, which excluded baseline comorbidities, yielded similar patterns, suggesting that the findings were not driven by adjustment for comorbidities (Table S8 in the **Online Supplementary Document**). Collectively, these results indicate that the combination of ADS initiation and high pack-years is associated with greater-than-additive increases in risk for COPD and chronic myocardial ischaemia, with the most pronounced interaction effect observed for COPD.

**Table 1:** Association of smoking initiation age with disease incidence and all-cause mortality stratified by pack-years*

	Total n	Events	HR (95% CI)†	Interaction *P*-value	RERI (95% CI)	AP (95% CI)
**Stroke**						
ADS	115,763	3,292	1.50 (1.40, 1.61)	0.076	0.01 (−0.40, 0.42)	0.01 (−0.31, 0.32)
Post-ADS	6,089	172	1.45 (1.05, 2.01)			
**Acute myocardial infarction**						
ADS	115,763	5,235	1.46 (1.38, 1.55)	0.095	0.12 (−0.18, 0.41)	0.10 (−0.17, 0.38)
Post-ADS	6,089	275	1.20 (0.92, 1.58)			
**Chronic myocardial ischaemia**						
ADS	115,763	11,687	1.38 (1.33, 1.44)	0.010	0.19 (0.00, 0.38)	0.17 (0.00, 0.34)
Post-ADS	6,089	568	1.08 (0.89, 1.30)			
**COPD**						
ADS	115,763	10,117	3.45 (3.29, 3.62)	<0.001	0.43 (0.08, 0.79)	0.16 (0.02, 0.29)
Post-ADS	6,089	442	2.33 (1.91, 2.83)			
**All-cause mortality**						
ADS	129,869	16,607	1.72 (1.66, 1.78)	<0.001	0.15 (−0.05, 0.34)	0.09 (−0.03, 0.22)
Post-ADS	6,659	742	1.57 (1.35, 1.83)			

ADS – alveolar developmental stage, AP – attributable proportion, CI – confidence interval, COPD – chronic obstructive pulmonary disease, HR – hazard ratio, RERI – risk due to interaction

*Adjusted covariates for all-cause mortality include demographics (age, sex, and education), lifestyle (alcohol and body mass index), environmental (nitrogen dioxide and particulate matter), comorbidities (angina, heart attack, stroke, hypertension, diabetes, and chronic kidney disease), and Townsend Deprivation Index; adjusted covariates for diseases include the same demographic/lifestyle/environmental factors, comorbidities (hypertension, diabetes, and chronic kidney disease), and Townsend Deprivation Index.

†Hazard ratios compare >20 *vs.* ≤20 pack-years.

### Smoking initiation during alveolar development increases the risk of comorbidities

Zero-inflated negative binomial regression revealed differential associations between heavy smoking (>20 pack-years) and comorbidity burden based on smoking initiation timing (**Table 2**). In the count model, which estimates the mean CCI score (adjusting for the probability of being in the zero-inflation group), heavy exposure was associated with a significantly higher comorbidity burden in both the ADS (incidence rate ratio = 1.24; 95% CI = 1.22, 1.27) and post-ADS (incidence rate ratio = 1.17; 95% CI = 1.05, 1.29) group. Although the effect appeared stronger in the ADS group, the interaction was not statistically significant (*P* for interaction = 0.129).

**Table 2:** ZINB regression associations of cumulative pack-year exposure (>20 pack-years *vs.* ≤20) with CCI by smoking initiation period (ADS *vs.* post-ADS)

	Count model*		Zero-inflation model†	
	**IRR (95% CI)**	**Interaction *P*-value**	**OR (95% CI)**	**Interaction *P*-value**
**ADS (n = 115,763)**	1.24 (1.22–1.27)	0.129	0.55 (0.52–0.59)	<0.001
**Post-ADS (n = 6,089)**	1.17 (1.05–1.29)		0.65 (0.43–0.97)	

ADS – alveolar developmental stage, CI – confidence interval, IRR – incidence rate ratio, OR – odds ratio, ZINB – zero-inflated negative binomial

*The count model presents incidence rate ratios for the expected Charlson Comorbidity Index score across the entire population, accounting for excess zeros. The model was adjusted for demographic (age, sex, and education), lifestyle (alcohol and body mass index), environmental (nitrogen dioxide and particulate matter), and Townsend Deprivation Index covariates.

†The zero-inflation model presents odds ratios for the probability of being in the ‘always zero’ or ‘structurally zero’ group, which in this context corresponds to having no comorbidities (Charlson Index = 0). The model was adjusted for demographic (age, sex, and education), lifestyle (alcohol and body mass index), environmental (nitrogen dioxide and particulate matter), and Townsend Deprivation Index covariates.

In the zero-inflation model, heavy smoking significantly reduced the probability of a zero CCI score. This reduction was significantly greater in the ADS (OR = 0.55; 95% CI = 0.52, 0.59) than in the post-ADS (OR = 0.65; 95% CI = 0.43, 0.97) group (*P* for interaction <0.001).

### Integrated smoking exposure score and population burden

**Figure 1:**
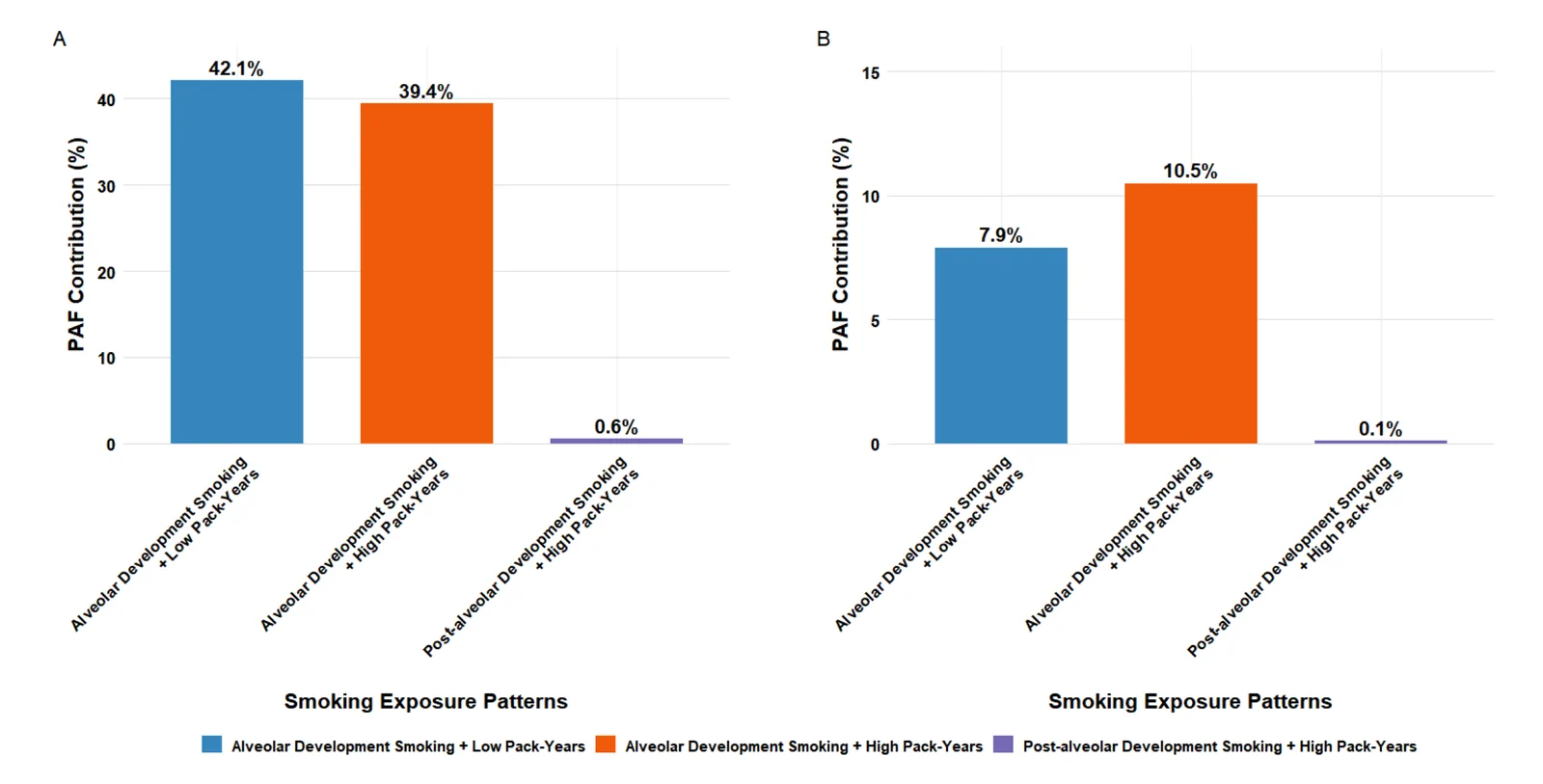
PAF of COPD cases and deaths associated with exposure score ≥1. **Panel A.** COPD. **Panel B.** All-cause mortality. COPD – chronic obstructive pulmonary disease. PAF – population-attributable fraction.

We developed a novel ordinal smoking exposure score (0–3 points) to establish biological risk hierarchies by integrating smoking initiation timing (ADS *vs.* post-ADS) and cumulative exposure dosage (≤20 *vs.* >20 pack-years). We defined the score categories as: 0 points (post-ADS initiation + low exposure; reference group), 1 point (post-ADS initiation + high exposure), 2 points (ADS initiation + low exposure), and 3 points (ADS initiation + high exposure). Multivariable-adjusted analyses showed each one-point score increment significantly elevated risks for COPD (HR = 2.29; 95% CI = 2.20, 2.38), stroke (HR = 1.24; 95% CI = 1.18–1.31), chronic myocardial ischaemia (HR = 1.18; 95% CI = 1.15–1.22), myocardial infarction (HR = 1.19; 95% CI = 1.14–1.25), and all-cause mortality (HR = 1.39; 95% CI = 1.36–1.43) (**Table 3**). Population-attributable fraction analysis indicated that 82.1% of COPD cases and 18.5% of deaths were attributable to exposures with score ≥1, with ADS initiation contributing most of the attributable burden (**Figure 1**, Panels A and B).

**Table 3:** Multivariable-adjusted associations of integrated smoking exposure score with cardiopulmonary outcomes and all-cause mortality*

	n/N	HR (95% CI)	*P*-value	Trend *P*-value
**COPD**	10,559/121,852	2.29 (2.20, 2.38)	<0.001	<0.001
**Stroke**	3,464/121,852	1.24 (1.18, 1.31)	<0.001	<0.001
**Chronic myocardial ischaemia**	12,255/121,852	1.18 (1.15, 1.22)	<0.001	<0.001
**Myocardial infarction**	5,510/121,852	1.19 (1.14, 1.25)	<0.001	<0.001
**All-cause mortality**	17,349/136,528	1.39 (1.36, 1.43)	<0.001	<0.001

CI – confidence interval, COPD – chronic obstructive pulmonary disease, CVD – cardiovascular disease, HR – hazard ratio

*Adjusted covariates include demographic (age, sex, and education), lifestyle (alcohol and body mass index), environmental (nitrogen dioxide and particulate matter), comorbidities (angina, heart attack, stroke, hypertension, diabetes, and chronic kidney disease), and Townsend Deprivation Index.

### The core value of the smoking exposure score in risk prediction

By integrating the alveolar developmental sensitive period and cumulative exposure levels, the smoking exposure score demonstrated pivotal value in predicting COPD and all-cause mortality risks. Time-dependent receiver operating characteristic curve analysis validated that incorporating the score significantly enhanced model discriminative efficiency, while decision curve analysis further demonstrated its clear net benefit advantage across clinically relevant risk thresholds (Figure S4 in the **Online Supplementary Document**). This scoring system effectively captures synergistic injury risks from developmental stage and cumulative exposure, which remain unrecognised by conventional indicators.

### ADS initiators gain greater long-term benefits from quitting

We found significantly steeper gradients of risk reduction for both all-cause mortality and COPD with prolonged smoking cessation among ADS initiators (*P* for interaction = 0.02) *vs.* post-ADS initiators (*P* for interaction <0.001) (Table S9 in the **Online Supplementary Document**). For all-cause mortality, ADS quitters demonstrated substantially lower adjusted HRs at >10 years cessation (HR = 0.42; 95% CI = 0.40, 0.43) compared to post-ADS quitters (HR = 0.51; 95% CI = 0.44, 0.60), with this advantage widening progressively across cessation intervals: ≤5 years (0.70 *vs.* 0.70) and 6–10 years (0.59 *vs.* 0.61). A parallel pattern was observed for COPD risk, where ADS quitters achieved superior protection at >10 years of cessation (HR = 0.24; 95% CI = 0.23, 0.25) *vs.* post-ADS quitters (HR = 0.35; 95% CI = 0.27, 0.46).

## DISCUSSION

We provide the first evidence of synergistic disease risk amplification between ADS initiation and cumulative tobacco exposure. Integrating NHANES and UKB data, we establish ADS initiation as an independent risk factor that potentiates per-unit exposure hazards for COPD, CVD, and all-cause mortality. We further developed a novel exposure stratification system quantifying joint risk hierarchies, revealing that ADS initiators exhibit disproportionately steeper gradients of relative risk reduction with prolonged smoking cessation compared to late initiators. Crucially, our findings indicate that while ADS initiators experience steeper rates of risk reduction upon cessation, their persistently elevated baseline vulnerability – likely from irreversible developmental insults – results in worse long-term outcomes compared to late initiators.

Extensive research has unequivocally established smoking as a major risk factor for numerous chronic diseases, which impose substantial social and economic burdens [24]. Substantial evidence confirms smoking's role as a risk factor for COPD and CVD, and several studies have explored the impact of single dimensions such as smoking status, cumulative dose, initiation age, or cessation duration [25–27]. However, the joint effect of smoking initiation age and cumulative exposure has never been systematically quantified, and how this developmental time window modifies the dose-response relationship of tobacco exposure remains unknown. Our study innovatively integrates exposure metrics (smoking initiation age and pack-years) to reveal, for the first time, the joint effect of smoking initiation age and pack-years on COPD risk.

The mechanisms underlying smoke-induced lung injury primarily involve oxidative stress and chronic inflammatory responses, leading to tissue destruction. For instance, reactive oxygen species and harmful chemicals in cigarette smoke can trigger lung cell apoptosis and DNA damage [28]. Regarding cardiovascular injury, mechanisms involve the activation of inflammatory signalling pathways mediated by smoke constituents (*e.g.* nicotine), resulting in endothelial dysfunction and early vascular stiffening, among other effects [29]. Studies suggest that exposure to smoke during critical developmental stages, as modelled in *Drosophila*, disrupts molecular processes when pulmonary and cardiovascular systems are forming, thereby increasing organ vulnerability and the subsequent risk of chronic diseases later in life [30]. This mechanistic insight provides a plausible explanation for the clinical observation of heightened disease risk in early-onset smokers, even at relatively low cumulative exposure levels.

Recent research indicates that individuals exposed to tobacco *in utero*, who initiate smoking in childhood and possess a high polygenic risk score, exhibit the most accelerated pace of biological ageing [31]. This acceleration of ageing may represent a key biological mechanism driving the development of multimorbidity. This is consistent with our findings that cumulative tobacco exposure during the critical alveolar developmental phase not only significantly elevates the risk of individual diseases but also substantially increases the probability of multimorbidity by accelerating systemic ageing processes. Our population attributable fraction analysis identifies smoke exposure during the alveolar development window as a core driver of the public health burden. This aligns with previous studies demonstrating that preventing early smoking initiation significantly reduces all-cause mortality and mortality burdens related to cancer and CVD [32]. Notably, we observed that among early initiators (those who began smoking during active alveolar development), prolonged smoking cessation was associated with a steeper relative risk reduction over time compared to late initiators. However, these findings are observational and must be interpreted with caution. Alternative explanations – including survivor bias, residual confounding, differences in smoking intensity trajectories, or age-related effects – cannot be ruled out, and causality should not be inferred. One speculative hypothesis that may generate future mechanistic research is that early-life smoke exposure during alveolarization could induce compensatory proliferative responses (*e.g.* AT2 cell hyperplasia) that, upon sustained cessation, might allow for greater relative functional recovery compared to late initiators. This hypothesis is supported by experimental studies of lung injury and repair [33,34]. Nevertheless, such a compensatory mechanism is likely constrained by the irreversible destruction of extracellular matrix components such as elastin and collagen fibres, which underlie emphysematous lesions [35]. From a public health perspective, these observational patterns suggest a potentially critical window for intervention: among adolescents who have already initiated smoking, proactive cessation efforts may yield substantial long-term mortality reductions, regardless of the underlying biology. Future studies integrating longitudinal imaging, biomarker assessments, or preclinical models will be necessary to test the biological plausibility of these speculative mechanisms.

### Strengths

This study has several strengths. First, we establish the ADS as a critical modifier of smoking dose-response relationships. Second, our dual-dimensional exposure scoring system – integrating developmental timing and cumulative dose – provides a clinically deployable tool that identifies high-risk profiles missed by conventional metrics, particularly early-life smokers with low cumulative exposure. Third, we document accelerated risk reduction trajectories following cessation in ADS smokers, creating an evidence base for prioritising adolescent cessation programmes irrespective of exposure duration.

### Limitations and future directions

Several limitations warrant consideration. First, smoking history relied on retrospective self-report, which may have introduced recall bias. Second, the NHANES analyses were cross-sectional and focused on prevalent outcomes, some of which were partly self-reported. Therefore, temporal sequence cannot be established, and these findings should not be interpreted as equivalent to the prospective incident-outcome analyses conducted in the UKB cohort. Third, although the UKB provided longitudinal follow-up and record-linked outcome ascertainment, its baseline age range (40–69 years) may limit generalisability to younger populations and may underestimate the full long-term consequences of early smoking initiation. Additionally, while the cutoff age of 24 years for the ADS was informed by prior literature and statistical optimisation, developmental timing is heterogeneous across individuals, and this threshold should therefore be interpreted as an operational definition rather than a precise biological boundary. Furthermore, the CCI, while widely used and weighted for severity, was originally designed for mortality prediction and may not fully capture the spectrum of smoking-related multimorbidity. Future studies could replicate the analysis using alternative multimorbidity measures. Finally, while the current model assumes a linear relationship between smoking exposure and risk, future research should explore nonlinear models to better capture the complexity of the interactions between smoking initiation age, cumulative exposure, and disease risk. Additionally, prospective birth or adolescent cohorts with repeated lung function assessments and detailed exposure data are needed to more accurately define developmental susceptibility windows and strengthen causal inferences. Lastly, although the smoking exposure score was designed as a risk stratification tool, external validation in independent cohorts is necessary to assess its clinical applicability and predictive power across diverse populations.

## CONCLUSIONS

Smoking initiation during ADS synergistically amplifies cumulative exposure effects, driving significantly elevated cardiopulmonary morbidity and overall comorbidity burden. Our novel dual-dimensional risk score stratifies exposure-disease hierarchies, identifying high-risk profiles overlooked by conventional metrics. Notably, while developmental insults may cause irreversible organ damage, ADS initiators show steeper relative risk reductions with prolonged smoking cessation compared to later initiators, suggesting a potentially critical window for youth-targeted cessation interventions before lung maturation. These findings support integrated strategies: preventing adolescent smoking initiation while actively promoting cessation in young smokers to maximise long-term health benefits.

## Additional material


Online Supplementary Document

## Data Availability

**Data availability:** This research has been conducted using the publicly available UKB and the NHANES datasets (https://www.cdc.gov/nchs/nhanes/index.html, https://www.ukbiobank.ac.uk).
