# Supplementary material for: Age-dependent health risks of smoking: greater harm associated with initiation before alveolar maturation (age 24)
Source: J Glob Health. 2026 Aug 28;16:04215. doi: 10.7189/jogh.16.04215 (PMC13521492; doi:10.7189/jogh.16.04215)
Supplement: Supplementary file 1 [file jogh-16-04215-s001.pdf]

Supplement to: Liu Z, Zhao Z, Xiao Y, Xiong R, Zheng H, Liu Y, Huang Q, Zhao R, Yan J, Chen Y, Zeng H. Age-dependent health risks of smoking: greater harm associated with initiation before alveolar maturation (age 24). J Glob Health. 2026;16:04215.

Table S1. Adherence to JoGH's GRABDROP guidelines items

**JoGH guideline items**	Our response
**1. Please list all papers published by each co-author in previous 3 years that were based on secondary analysis of a big data repository**	In the past three years, the co-authors have published the following papers based on secondary analyses of large public datasets-<br>1.Liu Z, Li J, Chen T, Zhao X, Chen Q, Xiao L, Peng Z, Zhang H. Association between dietary antioxidant levels and chronic obstructive pulmonary disease: a mediation analysis of inflammatory factors. Front Immunol. 2024 Jan 8;14:1310399. doi: 10.3389/fimmu.2023.1310399.<br>2.Liu Z, Su Y, Chen Q, Xiao L, Zhao X, Wang F, Peng Z, Zhang H. Association of Dietary intake of vitamin E with chronic obstructive pulmonary disease events in US adults: A cross-sectional study of NHANES 2013-2018. Front Nutr. 2023 Apr 12;10:1124648. doi: 10.3389/fnut.2023.1124648.<br>3.Li J, Liu Z, Pu Y, Dai H, Peng F. Association between dietary vitamin E intake and chronic kidney disease events in US adults: a cross-sectional study from NHANES 2009-2016. Clin Kidney J.2023 Jul 7;16(12):2559-2566. doi: 10.1093/ckj/sfad162.<br>4.Liu Z, Li J, Chen Q, Zhao X, Peng F, Zhang H. Associations of multiple metal intake with all-cause and cardiovascular mortality in US adults stratified by age and sex: A prospective cohort from the NHANES database study. J Trace Elem Med Biol.2024 May;83:127416. doi: 10.1016/j.jtemb.2024.127416.<br>5.Liu Z, Zeng H, Zhang H. Association of the oxidation balance score with the prevalence of chronic obstructive pulmonary disease from the NHANES 2007-2012: A large-scale cross-sectional study. Heart Lung. 2024 May-Jun;65:84-92. doi: 10.1016/j.hrtlng.<br>6.Li J, Liu Z, Xie X, Peng L, Dai H, Gao C, Mao W, Yuan W, Zhao X, Zhang H, Peng F. Dietary vitamin intake and cancer risk in patients with chronic kidney disease: results from the National Health and Nutrition Examination Survey (2007-2018). Int Urol Nephrol. 2024 Apr 26. doi: 10.1007/s11255-024-04060-2.
**2. Please explain the key elements of your study design and the use of the available datasets that make your study an original scientific contribution**	This study uses two large, complementary public datasets: NHANES (cross-sectional, US representative) and UK Biobank (prospective, UK). The original contributions are: (1) systematic quantification of multiplicative and additive interactions between smoking initiation age (≤24 vs. >24 years) and cumulative pack-years (>20 vs. ≤20) for COPD, CVD, mortality, and multimorbidity; (2) development of an ordinal smoking exposure score that integrates developmental timing and cumulative dose; (3) assessment of differential cessation benefits by initiation age. No prior study has jointly analysed these dimensions using both additive interaction metrics and prospective follow-up.

**JoGH guideline items**	Our response
3. Please list all publications that addressed similar research questions in the same dataset and indicate where you cited them in **your paper**	The following publications used the same datasets (UK Biobank or NHANES) to examine related but distinct questions, and they are cited in our manuscript as indicated:<br><br>Liu Z, Li J, Chen T, et al. Association between dietary antioxidant levels and chronic obstructive pulmonary disease: a mediation analysis of inflammatory factors. Front Immunol. 2023;14:1310399. (NHANES) – Cited as reference 20 in our manuscript. That study focused on dietary antioxidants and inflammatory mediators, not on smoking initiation timing or pack-year interactions.<br><br>Lin K, Miao Y, Gan L, et al. Associations of serum 25-hydroxyvitamin D concentrations with risks of mortality and cardiovascular disease among individuals with psoriasis. J Am Acad Dermatol. 2025. (UK Biobank) – Cited as reference 21. That study examined vitamin D in psoriasis patients, a different exposure and population.<br>Hanlon P, Jani BD, Butterly E, et al. An analysis of frailty and multimorbidity in 20,566 UK Biobank participants with type 2 diabetes. Commun Med (Lond). 2021;1:28. (UK Biobank) – Cited as reference 22. That study analysed frailty and multimorbidity specifically in diabetic patients, not smoking-related outcomes.
**4. Please explain how you addressed multiple testing through an appropriately rigorous statistical threshold and indicate this in the methods section**	We pre-specified a limited set of primary outcomes (COPD, cardiovascular disease, all-cause mortality, and multimorbidity) and two types of interaction tests (multiplicative and additive) for each outcome, resulting in fewer than 10 primary statistical tests (specifically, 4 outcomes × 2 interaction types = 8 primary tests). For these confirmatory analyses, we did not adjust the significance threshold for multiple comparisons because: (1) all primary findings had p-values <0.001, which would remain statistically significant even after a conservative Bonferroni correction (e.g., $\alpha=0.05/8=0.00625$); (2) the number of primary tests was small and pre-specified. For exploratory secondary analyses (e.g., subgroup analyses by sex, specific CVD subtypes), we report exact p-values without formal adjustment and interpret them as hypothesis-generating. This approach is explicitly stated in the Methods section (Statistical Analysis).
**5. Please declare to what extent have AI chatbots been used in developing your paper and to which parts of the paper did they contribute**	AI chatbots (specifically, a large language model, ChatGPT) were used only for minor language polishing and rephrasing of certain sentences to improve clarity and conciseness. No AI was used for data analysis, interpretation, or generation of scientific conclusions. The original statistical code, results, tables, figures, and the core scientific argument were entirely produced by the human authors.

Table S2.Comparison of COPD Risk Between Smoking Exposure During vs. After Alveolar Development Period: Hazard Ratios (HR) Risk Differences (Δ HR) and Interaction Significance

Evaluation Metric	15-year Cutoff	19-year Cutoff	24-year Cutoff	Advantage of 24-year
**Risk Difference (HR)**	3.29 vs 3.25	3.50 vs 2.80	3.45 vs 2.33	Largest risk difference (Δ1.12)
**Interaction Effect**	p=0.862	p<0.001	P<0.001	Highly significant interactio

Table S3. Sensitivity analysis: Smoking initiation age as a continuous variable and risk of COPD

Model	HR (per 1-year increase)	95% CI	p value
**Basic model***	0.990	0.985 – 0.995	0.000165
**Fully adjusted model†**	0.990	0.984 – 0.995	0.000134

***Adjusted for sex, age, pack-years, education, alcohol, BMI, NO_2, PM2.5, and Townsend index.**
†Further adjusted for hypertension, diabetes, and chronic kidney disease

Table S4. Disease Characteristics of NHANES Participants by Smoking Initiation Age Group

Characteristic	Overall	Never smoked	Alveolar development	Post-alveolar development	P-value
**n**	15077	8627	5859	591	
**Age years**	47.29 ± 16.63	45.91 ± 16.70	48.75 ± 16.41	54.57 ± 14.58	**<0.001**
**Smoking index**	160.61 ± 360.13	0.00 ± 0.00	394.26 ± 482.18	209.43 ± 288.69	**<0.001**
**Sex n (%)**					**<0.001**
Female	7642 (51.3)	5019 (56.5)	2320 (43.3)	303 (54.7)	
Male	7435 (48.7)	3608 (43.5)	3539 (56.7)	288 (45.3)	
**Race n (%)**					**<0.001**
Mexican American	2331 (8.2)	1521 (9.5)	707 (6.1)	103 (9.5)	
Other	2874 (12.3)	1915 (14.3)	848 (9.3)	111 (13.8)	
NonHispanic White	6795 (68.9)	3395 (64.9)	3182 (75.4)	218 (61.1)	

Characteristic	Overall	Never smoked	Alveolar development	Post-alveolar development	P-value
NonHispanic Black	3077 (10.6)	1796 (11.3)	1122 (9.2)	159 (15.6)	
**Marital status n (%)**					**<0.001**
Married/cohabiting	8965 (63.8)	5130 (64.1)	3535 (64.2)	300 (54.6)	
Divorced/separated/widowed	3399 (18.2)	1740 (15.9)	1440 (20.5)	219 (33.7)	
Never married	2713 (17.9)	1757 (20.0)	884 (15.3)	72 (11.7)	
**Education n (%)**					**<0.001**
< High school	4120 (18.3)	2081 (14.7)	1857 (23.0)	182 (23.3)	
High school graduate	3423 (22.4)	1783 (20.0)	107 (26.1)	133 (22.4)	
> High school	7534 (59.3)	4763 (65.3)	2495 (50.9)	276 (54.2)	
**BMI n (%)**					**0.588**
≤25 kg/m²	4433 (31.3)	2516 (31.0)	1754 (32.1)	163 (28.4)	
25-30 kg/m²	5140 (34.2)	2925 (34.4)	2013 (34.1)	202 (33.8)	
>30 kg/m²	5504 (34.4)	3186 (34.6)	2092 (33.9)	226 (37.8)	
**Alcohol status n (%)**					**<0.001**
Never	2044 (10.3)	1778 (15.7)	216 (2.8)	50 (6.0)	
Former	2691 (14.9)	1241 (12.1)	1316 (18.8)	134 (19.4)	
Light-moderate	7253 (53.4)	4323 (56.6)	2640 (48.6)	290 (52.8)	
Heavy	3089 (21.3)	1285 (15.6)	1687 (29.8)	117 (21.8)	
**Poverty-income ratio n (%)**					**<0.001**
≤1	3090 (14.1)	1574 (12.4)	176 (16.4)	140 (18.3)	
1-3	6730 (36.9)	3762 (35.1)	2677 (39.1)	291 (43.9)	
>3	5257 (48.9)	3291 (52.6)	1806 (44.5)	160 (37.7)	

Characteristic	Overall	Never smoked	Alveolar development	Post-alveolar development	P-value
**Cardiovascular disease n (%)**					**<0.001**
No	13453 (91.5)	7947 (93.8)	5011 (88.6)	495 (85.3)	
Yes	1624 (8.5)	680 (6.2)	848 (11.4)	96 (14.7)	
**Chronic kidney disease n (%)**					**0.001**
No	12346 (86.1)	7209 (87.2)	4684 (84.8)	453 (82.0)	
Yes	2731 (13.9)	1418 (12.8)	1175 (15.2)	138 (18.0)	
**Diabetes n (%)**					**<0.001**
No	12271 (86.5)	7180 (88.1)	4637 (84.7)	454 (81.5)	
Yes	2806 (13.5)	1447 (11.9)	1222 (15.3)	137 (18.5)	
**Hypertension n (%)**					**<0.001**
No	7163 (51.9)	4370 (54.8)	2577 (48.7)	216 (39.4)	
Yes	7914 (48.1)	4257 (45.2)	3282 (51.3)	375 (60.6)	
**COPD n (%)**					**<0.001**
No	14165 (93.9)	8440 (97.7)	5190 (88.3)	535 (90.7)	
Yes	912 (6.1)	187 (2.3)	669 (11.7)	56 (9.3)	
**Stroke n (%)**					**<0.001**
No	14456 (96.9)	8377 (97.6)	5524 (95.9)	555 (95.0)	
Yes	621 (3.1)	250 (2.4)	335 (4.1)	36 (5.0)	
**Angina n (%)**					**<0.001**

Characteristic	Overall	Never smoked	Alveolar development	Post-alveolar development	P-value
No	14717 (98.0)	8471 (98.5)	5679 (97.4)	567 (95.9)	
Yes	360 (2.0)	156 (1.5)	180 (2.6)	24 (4.1)	
**Myocardial infarction n (%)**					**<0.001**
No	14424 (96.6)	8389 (97.9)	5480 (95.0)	555 (94.7)	
Yes	653 (3.4)	238 (2.1)	379 (5.0)	36 (5.3)	
**Liver cirrhosis n (%)**					**<0.001**
No	11115 (78.1)	6557 (80.1)	4152 (75.8)	406 (71.7)	
Yes	3962 (21.9)	2070 (19.9)	1707 (24.2)	185 (28.3)	

Note:COPD: Chronic obstructive pulmonary disease,BMI,Body Mass Index;Categorical variables were presented as number of persons before weighting (weighted percentages). Continuous variables were presented as weighted means ± standard deviation.

Table S5. Disease Characteristics of UK Biobank Participants by Smoking Initiation Age Group

Characteristic	Alveolar development(n=115763)	Post-alveolar development (n=6089)	P-value
**Age years**	58.0 (51.0 63.0)	58.0 (49.0 64.0)	0.003
**BMI kg/m²**	27.2 (24.5 30.3)	26.9 (24.1 30.1)	<0.001
**NO_2 µg/m³**	26.6 (21.8 31.7)	28.2 (22.9 33.5)	<0.001
**PM2.5-10 µg/m³**	6.1 (5.8 6.7)	6.2 (5.9 6.7)	<0.001
**TOWNSEND deprivation index**	-1.8 (-3.4 1.1)	-0.9 (-3.0 2.2)	<0.001
**Sex n (%)**			<0.001
Male	59108 (51.1)	2363 (38.8)	

Characteristic	Alveolar development(n=115763)	Post-alveolar development (n=6089)	P-value
Female	56655 (48.9)	3726 (61.2)	
**Education n (%)**			<0.001
Other	86238 (74.5)	4121 (67.7)	
College/University	29525 (25.5)	1968 (32.3)	
**Alcohol status n (%)**			<0.001
Never	1928 (1.7)	184 (3.0)	
Previous	5204 (4.5)	326 (5.4)	
Current	108631 (93.8)	5579 (91.6)	
**Hypertension n (%)**			0.005
No	86352 (74.6)	4639 (76.2)	
Yes	29411 (25.4)	1450 (23.8)	
**Diabetes n (%)**			0.140
No	109134 (94.3)	5768 (94.7)	
Yes	6629 (5.7)	321 (5.3)	
**Chronic kidney disease n (%)**			0.078
No	114814 (99.2)	6052 (99.4)	
Yes	949 (0.8)	37 (0.6)	
**pack-years n (%)**			<0.001
<=20	63392 (54.8)	4826 (79.3)	
>20	52371 (45.2)	1263 (20.7)	
**COPD n (%)**			<0.001
No	105646 (91.3)	5647 (92.7)	
Yes	10117 (8.7)	442 (7.3)	

Characteristic	Alveolar development(n=115763)	Post-alveolar development (n=6089)	P-value
**Heart failure n (%)**			0.054
No	104076 (89.9)	5521 (90.7)	
Yes	11687 (10.1)	568 (9.3)	
**Stroke n (%)**			0.968
No	112471 (97.2)	5917 (97.2)	
Yes	3292 (2.8)	172 (2.8)	
**Myocardial infarction n (%)**			1.000
No	110528 (95.5)	5814 (95.5)	
Yes	5235 (4.5)	275 (4.5)	

Note:COPD: Chronic obstructive pulmonary disease,BMI,Body Mass Index;NO2,Nitrogen Dioxide;PM2.5-10,Particulate Matter 2.5 to 10 micrometers.Categorical variables were presented as numbers (percentages). Continuous variables were presented as medians (Q1 Q3).

Table S6. Mortality Outcomes in UK Biobank by Smoking Initiation Age

Characteristic	Alveolar development(**n=129869)**	Post-alveolar development **(n=6659)**	P-value
**Age years**	59.0 (52.0 64.0)	59.0 (50.0 64.0)	0.001
**BMI kg/m²**	27.3 (24.6 30.5)	27.0 (24.2 30.3)	<0.001
**NO_2 μg/m³**	26.7 (21.9 31.7)	28.2 (23.0 33.6)	<0.001
**PM2.5-10 μg/m³**	6.1 (5.8 6.7)	6.2 (5.9 6.7)	<0.001
**TOWNSEND deprivation index**	-1.7 (-3.4 1.3)	-0.8 (-3.0 2.3)	<0.001
**Sex n (%)**			<0.001
Male	68910 (53.1)	2674 (40.2)	
Female	60959 (46.9)	3985 (59.8)	

Characteristic	Alveolar development(n=129869)	Post-alveolar development (n=6659)	P-value
**Education n (%)**			<0.001
Other	98276 (75.7)	4569 (68.6)	
College/University	31593 (24.3)	2090 (31.4)	
**Alcohol status n (%)**			<0.001
Never	2333 (1.8)	214 (3.2)	
Previous	6330 (4.9)	392 (5.9)	
Current	121206 (93.3)	6053 (90.9)	
**Hypertension n (%)**			<0.001
No	94088 (72.4)	4960 (74.5)	
Yes	35781 (27.6)	1699 (25.5)	
**Diabetes n (%)**			0.218
No	120945 (93.1)	6228 (93.5)	
Yes	8924 (6.9)	431 (6.5)	
**Chronic kidney disease n (%)**			0.018
No	128674 (99.1)	6617 (99.4)	
Yes	1195 (0.9)	42 (0.6)	
**Angina pectoris n (%)**			<0.001
No	123886 (95.4)	6424 (96.5)	
Yes	5983 (4.6)	235 (3.5)	
**Myocardial infarction n (%)**			<0.001
No	124839 (96.1)	6463 (97.1)	
Yes	5030 (3.9)	196 (2.9)	
**Stroke n (%)**			0.412

Characteristic	Alveolar development(n=129869)	Post-alveolar development (n=6659)	P-value
No	127192 (97.9)	6532 (98.1)	
Yes	2677 (2.1)	127 (1.9)	
**All-cause mortality n (%)**			<0.001
No	113262 (87.2)	5917 (88.9)	
Yes	16607 (12.8)	742 (11.1)	
Pack-years n (%)			<0.001
0-20	67542 (52.0)	5136 (77.1)	
>20	62327 (48.0)	1523 (22.9)	

Note:COPD: Chronic obstructive pulmonary disease,BMI,Body Mass Index;NO2,Nitrogen Dioxide;PM2.5-10,Particulate Matter 2.5 to 10 micrometers.Categorical variables were presented as numbers (percentages). Continuous variables were presented as medians (Q1 Q3)

Table S7. Association between Smoking Initiation Age and Diseases in NHANES

Disease	Variable	OR (95% CI)	p-value
COPD	Alveolar development	3.48 (2.72–4.44)	< 0.001
	Post-alveolar development	2.79 (1.95–3.98)	0.001
CKD	Alveolar development	0.94 (0.78–1.13)	0.493
	Post-alveolar development	0.82 (0.58–1.16)	0.256
Diabetes	Alveolar development	1.08 (0.89–1.32)	0.416
	Post-alveolar development	1.02 (0.78–1.33)	0.884
Hypertension	Alveolar development	1.07 (0.96–1.20)	0.210
	Post-alveolar development	1.16 (0.88–1.54)	0.288
Angina	Alveolar development	1.00 (0.69–1.45)	0.989
	Post-alveolar development	1.29 (0.62–2.69)	0.486
Coronary heart disease	Alveolar development	1.15 (0.84–1.57)	0.388

Disease	Variable	OR (95% CI)	p-value
	Post-alveolar development	1.91 (1.01–3.62)	0.047
Heart attack	Alveolar development	1.48 (1.05–2.08)	0.026
	Post-alveolar development	0.99 (0.49–2.03)	0.987
Stroke	Alveolar development	1.41 (1.06–1.86)	0.018
	Post-alveolar development	1.21 (0.71–2.06)	0.473
Liver cirrhosis	Alveolar development	0.97 (0.80–1.61)	0.703
	Post-alveolar development	0.77 (0.48–1.24)	0.283

Note:COPD: Chronic obstructive pulmonary disease; CKD: Chronic kidney disease; OR: Odds ratio; CI: Confidence interval.

Table S8. Association of smoking initiation age with disease incidence and all-cause mortality stratified by pack-years

Outcome	Smoking Initiation Age Group	n	Events	Hazard Ratio (95% CI)	P for interaction	RERI (95% CI)	AP (95% CI)
Stroke	Alveolar development	115,763	3,292	1.52 (1.42–1.64)	0.081	-0.01 (-0.43, 0.41)	-0.01 (-0.32, 0.31)
	Post-Alveolar development	6,089	172	1.46 (1.06–2.03)			
Acute Myocardial Infarction	Alveolar development	115,763	5,235	1.48 (1.40–1.57)	0.116	0.11 (-0.19, 0.41)	0.10 (-0.17, 0.37)

Outcome	Smoking Initiation Age Group	n	Events	Hazard Ratio (95% CI)	P for interaction	RERI (95% CI)	AP (95% CI)
	Post-Alveolar development	6,089	275	1.22 (0.93–1.60)			
Chronic Myocardial Ischemia	Alveolar development	115,763	11,687	1.41 (1.36–1.46)	0.0145	0.19 (0.00, 0.38)	0.16 (0.00, 0.34)
	Post-Alveolar development	6,089	568	1.10 (0.91–1.32)			
COPD	Alveolar development	115,763	10,117	3.48 (3.32–3.64)	<0.001	0.42 (0.07, 0.78)	0.15 (0.02, 0.29)
	Post-Alveolar development	6,089	442	2.35 (1.93–2.86)			
All-cause mortality	Alveolar development	129,869	16,607	1.80 (1.74–1.86)	*<0.001	0.14 (-0.06, 0.34)	0.09 (-0.04, 0.21)
	Post-Alveolar development	6,659	742	1.60 (1.37–1.86)			

Notes: HR, hazard ratio; CI, confidence interval; RERI, relative excess risk due to interaction; AP, attributable proportion due to interaction. Hazard ratios compare >20 vs. ≤20 pack-years within each smoking initiation age group. Adjusted for sex, age, education, alcohol, BMI, NO_2, $PM_{2.5-10}$, and Townsend deprivation index (baseline comorbidities not included).

Table S9. Mortality and COPD Risk by Alveolar Development Stage and Smoking Cessation

Outcome	Variable	Alveolar Development HR (95% CI)	Post-Alveolar Development HR (95% CI)	P for interaction
**All-cause mortality**				0.02
	Total (Events)	128,955 (16,411)	7,573 (938)	-
	Quit ≤5 years	0.70 (0.66–0.74)	0.70 (0.57–0.86)	
	Quit 6–10 years	0.59 (0.55–0.62)	0.61 (0.49–0.77)	
	Quit >10 years	0.42 (0.40–0.43)	0.51 (0.44–0.60)	
**COPD**				**<0.001**
	Total (Events)	115,763 (10,117)	6,089 (442)	
	Quit ≤5 years	0.51 (0.48–0.54)	0.54 (0.41–0.73)	
	Quit 6–10 years	0.42 (0.39–0.45)	0.48 (0.35–0.67)	
	Quit >10 years	0.24 (0.23–0.25)	0.35 (0.27–0.46)	

Note: COPD: Chronic obstructive pulmonary disease. Reference group: Current smokers in all analyses. Adjusted covariates for all-cause mortality: demographic (age, sex, education), lifestyle (alcohol, BMI), environmental (PM2.5-10, NO_2), comorbidities (angina, myocardial infarction, stroke, hypertension, diabetes, CKD), cigarette pack-years, and Townsend deprivation index. For COPD: same demographic/lifestyle/environmental factors, cigarette pack-years, and Townsend deprivation index; comorbidities (hypertension, diabetes, CKD).

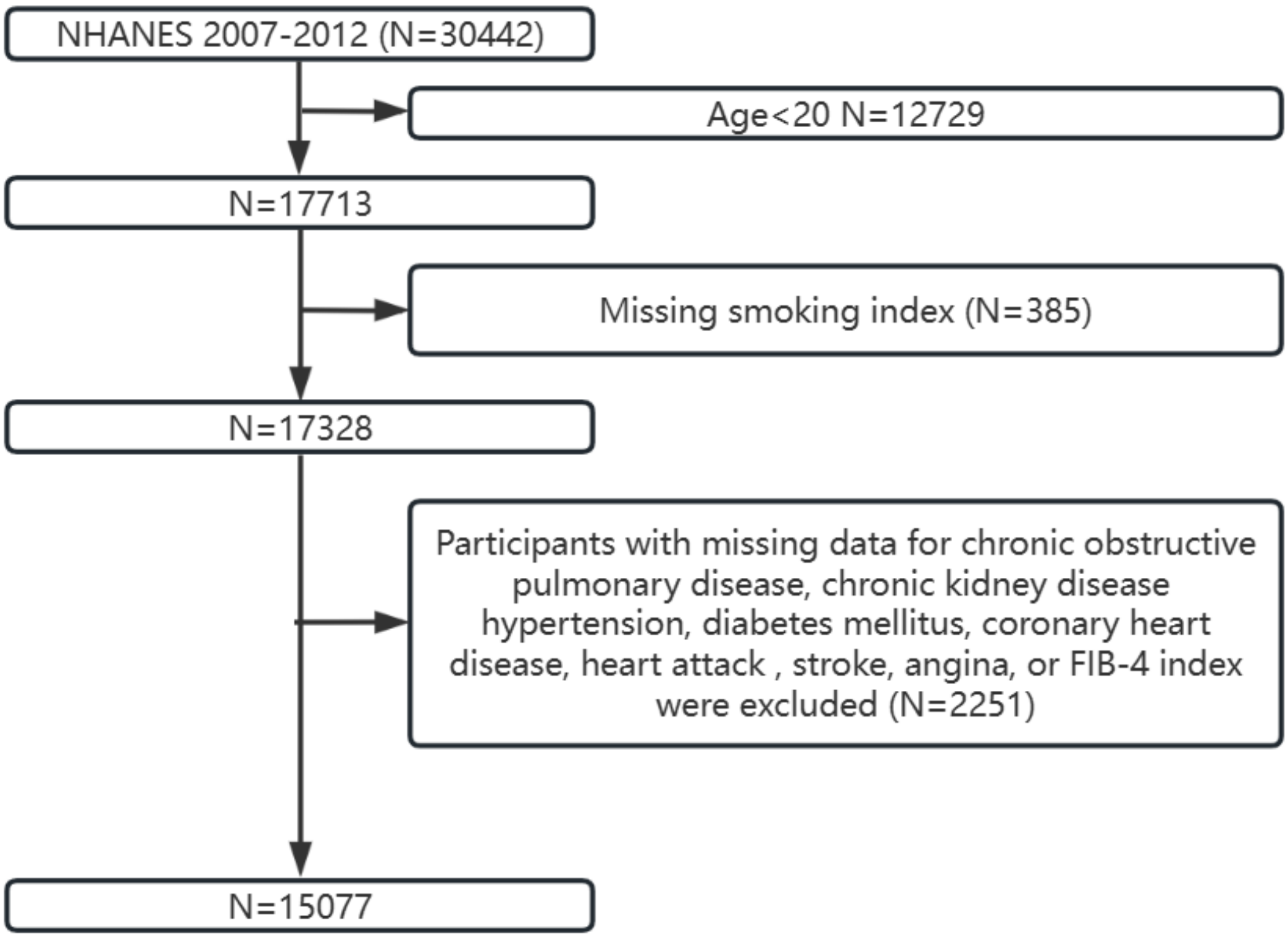

Figure S1. Flowchart of NHANES Population Screening

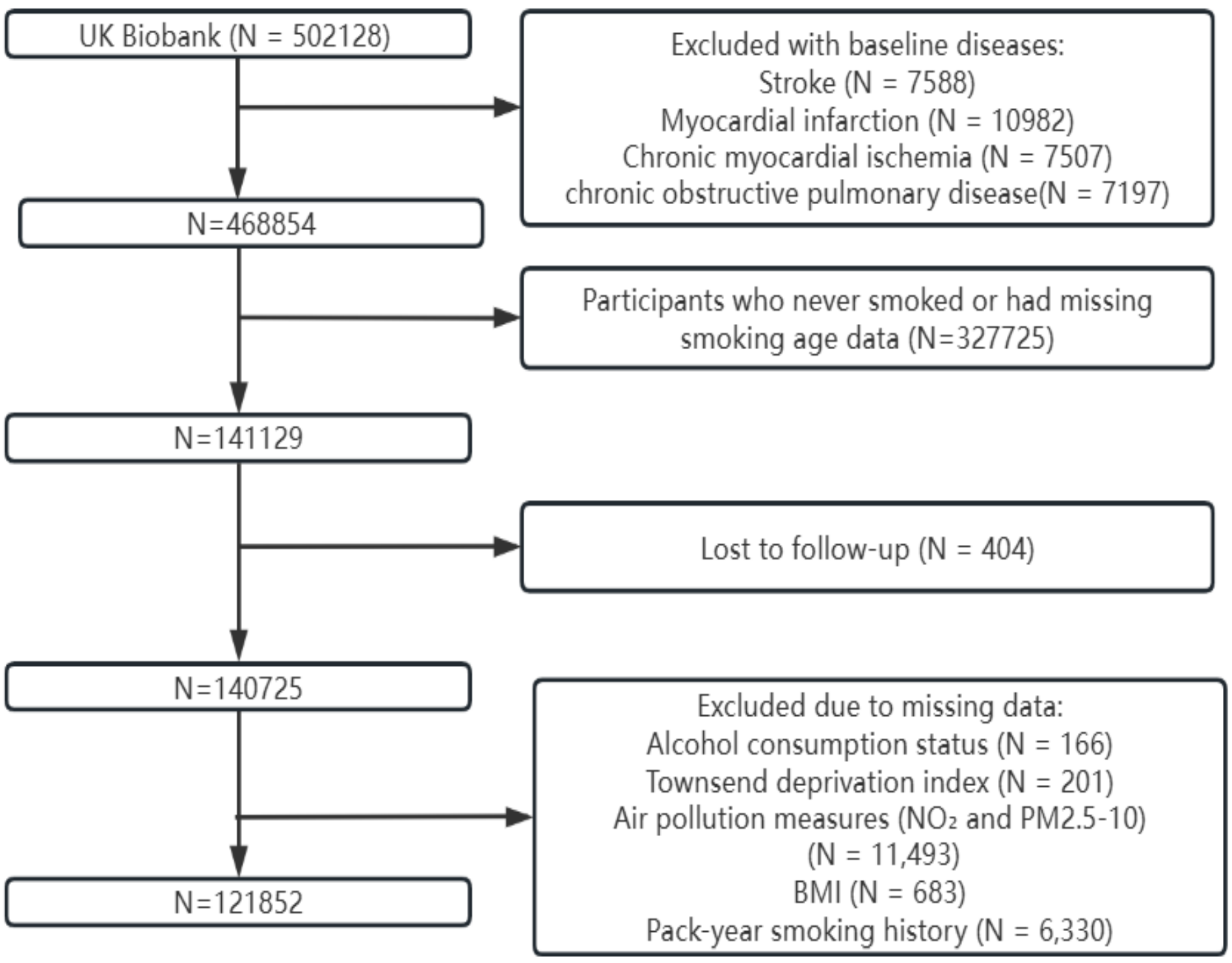

Figure S2. Flowchart of Population Screening for UK Biobank Disease Cohort

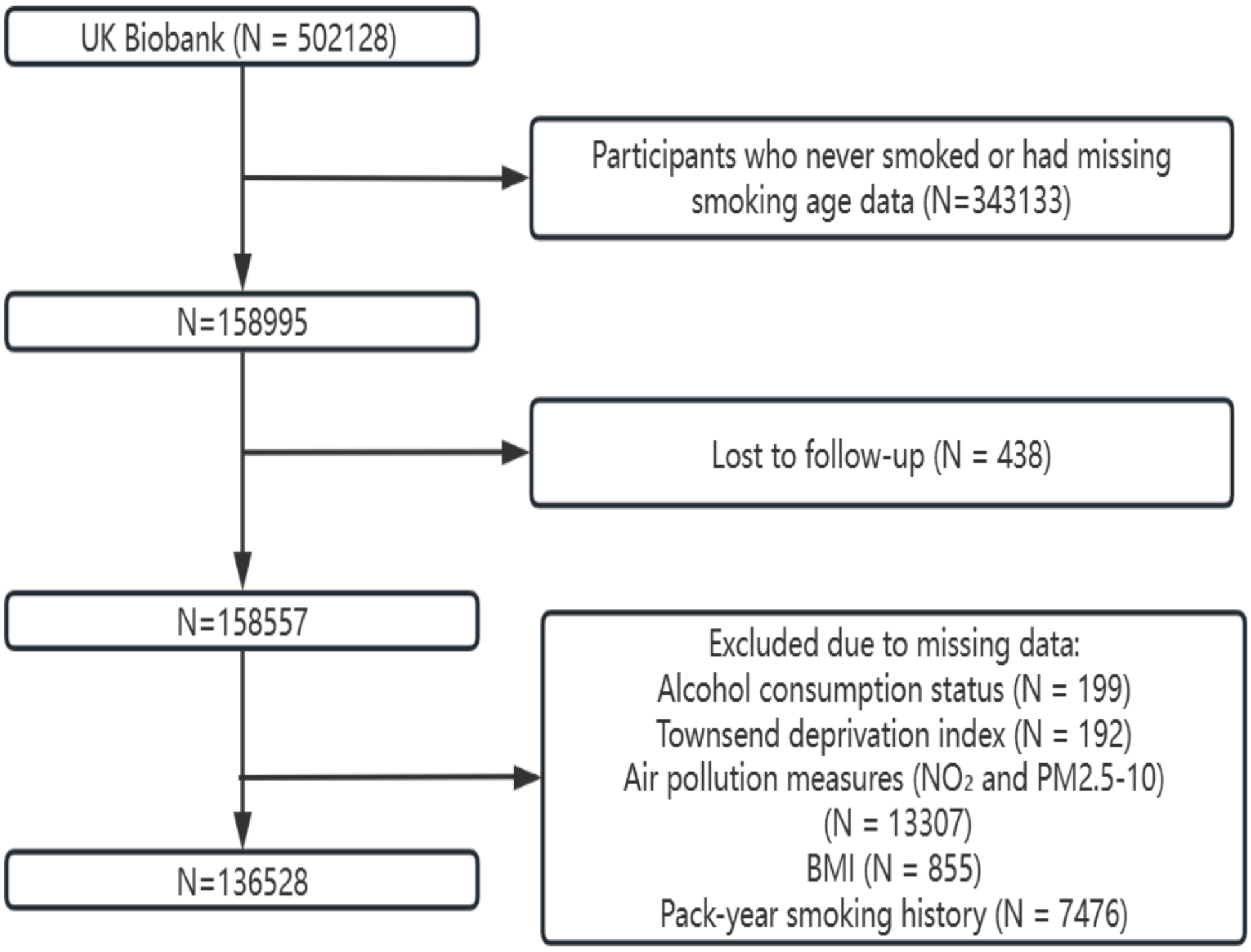

Figure S3. Flowchart of Population Screening for UK Biobank Mortality Cohort

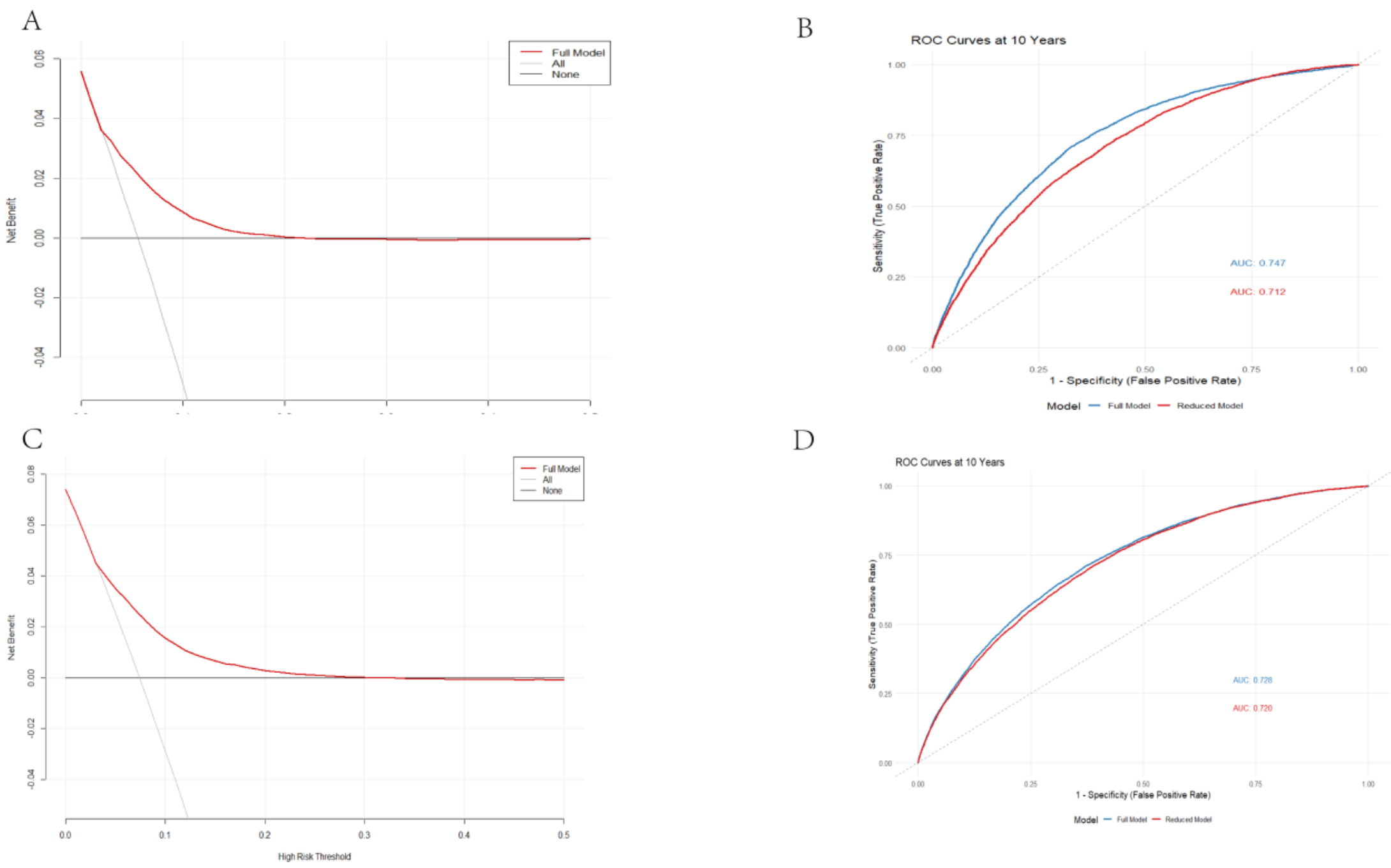

Figure S4. DCA and ROC Analyses of the Risk Score for Predicting COPD and All-Cause Mortality

(A) DCA for COPD (B) Time-dependent ROC curve for COPD (C) DCA for all-cause mortality (D) Time-dependent ROC curve for all-cause mortality
